# STAT1 coordinates intestinal epithelial cell death during gastrointestinal infection upstream of Caspase-8

**DOI:** 10.1038/s41385-021-00450-2

**Published:** 2021-09-08

**Authors:** Iris Stolzer, Laura Schickedanz, Mircea T. Chiriac, Rocío López-Posadas, Guntram A. Grassl, Jochen Mattner, Stefan Wirtz, Beate Winner, Markus F. Neurath, Claudia Günther

**Affiliations:** 1 Department of Medicine 1, Universitätsklinikum Erlangen and Friedrich-Alexander-Universität (FAU), Erlangen, Germany; 2 Deutsches Zentrum Immuntherapie DZI, Universitätsklinikum Erlangen and Friedrich-Alexander-Universität (FAU), Erlangen, Germany; 3 Institute of Medical Microbiology and Hospital Epidemiology, Hannover Medical School and German Center for Infection Research (DZIF), Hannover, Germany; 4 Mikrobiologisches Institut-Klinische Mikrobiologie, Immunologie und Hygiene, Universitätsklinikum Erlangen and Friedrich-Alexander-Universität (FAU), Erlangen, Germany; 5 Department of Stem Cell Biology, Universitätsklinikum Erlangen and Friedrich-Alexander-Universität (FAU), Erlangen, Germany; 6 Center for Rare Diseases Erlangen (ZSEER), Universitätsklinikum Erlangen, Erlangen, Germany

## Abstract

Intestinal homeostasis and the maintenance of the intestinal epithelial barrier are essential components of host defense during gastrointestinal *Salmonella* Typhimurium infection. Both require a strict regulation of cell death. However, the molecular pathways regulating epithelial cell death have not been completely understood. Here, we elucidated the contribution of central mechanisms of regulated cell death and upstream regulatory components during gastrointestinal infection. Mice lacking Caspase-8 in the intestinal epithelium are highly sensitive towards bacterial induced enteritis and intestinal inflammation, resulting in an enhanced lethality of these mice. This phenotype was associated with an increased STAT1 activation during *Salmonella* infection. Cell death, barrier breakdown and systemic infection were abrogated by an additional deletion of STAT1 in *Casp8*^ΔIEC^ mice. In the absence of epithelial STAT1, loss of epithelial cells was abolished which was accompanied by a reduced Caspase-8 activation. Mechanistically, we demonstrate that epithelial STAT1 acts upstream of Caspase-8-dependent as well as -independent cell death and thus might play a major role at the crossroad of several central cell death pathways in the intestinal epithelium. In summary, we uncovered that transcriptional control of STAT1 is an essential host response mechanism that is required for the maintenance of intestinal barrier function and host survival.

## Introduction

The invasive bacterium *Salmonella enterica* is a common gastrointestinal pathogen that causes severe foodborne illness in humans worldwide^1^. Infection with *Salmonella enterica* serovar Typhimurium leads to acute, self-limiting intestinal inflammation associated with diarrhea, vomiting and abdominal pain^2^. The ability of pathogens such as *Salmonella* to colonize and invade the gut is controlled by several factors including the commensal microflora, the immune system, and the intestinal epithelial barrier^3^. To gain access to the underlying immune system and host circulation, pathogens have to conquer the epithelium. Thus, infection with *Salmonella* is characterized by increased cell death within the mucosal tissue and particularly in the intestinal epithelium during early infection. Whether epithelial cell death represents a host defense mechanism during gastrointestinal infection or whether it is a bacterial strategy to colonize and persist in the host is controversially discussed^4^. In the context of *Salmonella* Typhimurium, gastrointestinal infection is accompanied by epithelial cell death with a complex underlying signaling cascade and associated with the upregulation of several key members of central cell death pathways. These include, but are not limited to *Caspase-8* and *Caspase-3* involved in apoptosis, increased *Caspase-1* and *IL-1b* expression controlling pyroptosis and elevated *mixed lineage kinase domain like pseudokinase* (*Mlkl*) levels associated with necroptosis^5–9^. Consequently, this complex molecular network requires regulatory mechanisms that coordinate these different cell death pathways to ensure pathogen clearance and host survival.

One central cell death regulator is Caspase-8. Previous studies have demonstrated that epithelial Caspase-8 is essential to promote host resistance to *Salmonella* Typhimurium by orchestrating mucosal defense mechanisms to control bacterial burden and to prevent tissue colonization^6^. Furthermore, Caspase-8 is implicated in intestinal cell expulsion by NLRC4 inflammasome activation^8^. Novel studies further linked a complex cell death signaling cascade involving apoptosis, pyroptosis and necroptosis and thus denoted as PANoptosis, to gastrointestinal infection^10^. Pyroptosis, is characterized by elevated levels of Caspase-1 and/or Caspase-11 as well as inflammasome activation and is driven by pore-formation mediated by Gasdermin D (GSDMD). Similarly, necroptosis is executed by plasma membrane nanopores driven by MLKL oligomerization^11^. We have previously demonstrated that negative regulation of this process by Caspase-8 (using *Casp8*^ΔIEC^ mice, lacking Caspase-8 in the intestinal epithelium) plays an essential role during enteric infection^6^. Accordingly, we uncovered that the lack of this control mechanism is associated with a lethal outcome of *Casp8*^ΔIEC^ mice after infection with *S*. Typhimurium due to excessive epithelial cell death during the early phase of infection. Equal *Mlkl* expression levels during infection observed in control and *Casp8*^ΔIEC^ mice highlights the role of Caspase-8 as central cell death regulator to prevent massive and harmful necroptosis. Interestingly, lethality of *Casp8*^ΔIEC^ mice only partially depends on TNF^6^. Hence, it has been shown that beside TNF, interferons (IFNs) can trigger epithelial cell death by activating the STAT1 pathway^12,13^ and increased interferon expression was described during *Salmonella* infection for *Casp8*^ΔIEC^ mice^6^.

Interferons including type I, type II as well as type III, are associated with cell death regulation and pathogen control. Accordingly, type I interferon-induced necroptosis in macrophages was associated with immune escape and bacterial spread^14–17^. Type II IFN signaling restricts pathogen load in the mucosal tissue by enhancing the production and release of mucus by goblet cells as well as secretion of antimicrobial peptide filled vacuoles from Paneth cells^18,19^. Furthermore, type II IFN prevents bacterial spread in the gut by triggering the release of the bacteria from the *Salmonella-*containing vacuole into the cytosol of infected cells^5^. In addition, also type III interferons display host protective effects by preventing transcellular bacterial spread through stabilization of epithelial barrier integrity beside its cell-intrinsic antiviral functions^20^.

On a molecular level, IFNs trigger via their respective receptors the Janus kinase (JAK) - signal transducer and activator of transcription (STAT) signaling pathway. The complex JAK-STAT pathway can induce a variety of genes depending on the cellular and disease context and it is not surprising that these cytokines can be friend or foe during infection^21–23^. Accordingly, studies in mice and humans reveal that subjects with mutations in *STAT1* are susceptible to infection by viral and bacterial pathogens, highlighting the essential role of STAT1 during infection^24–29^. However, the full scope of the interferon-STAT1 signaling cascade on cell death regulation and maintenance of intestinal homeostasis is not fully understood.

Here, we demonstrate that epithelial STAT1 signaling is essential for host defense during bacterial enteritis by controlling cell extrusion in the intestinal epithelium. On a molecular level, we uncovered that the transcription factor STAT1 induces gene expression of several key members of central cell death pathways and controls the activation of Caspase-8-dependent and -independent cell death in the intestinal epithelium during gastrointestinal infection. This is particularly important during the initial phase of infection for orchestrating cellular extrusion of infected epithelial cells. Notably, this STAT1 mediated activation of cell death seems to be a widespread control mechanism for a variety of pathogens including intracellular as well as attaching bacteria. Collectively, our study reveals that STAT1-signaling is essential to coordinate regulated epithelial cell death and that a disruption of this signaling pathway disturbs mucosal homeostasis during *Salmonella* infection.

## Results

### STAT1 coordinates epithelial cell death during *Salmonella* induced enteritis

Previously, we have shown that Caspase-8 plays a key role during *Salmonella* infection by mediating epithelial apoptosis and inhibition of programmed necrosis (necroptosis) (Supplementary Fig. S1A)^6^. Lack of Caspase-8 in the intestinal epithelium triggers excessive necroptosis associated with the breakdown of the intestinal barrier, which finally results in systemic bacterial spread. Massive inflammatory cell death is concomitant with an enhanced pro-inflammatory cytokine expression, which promotes further cell death by transcriptional regulation and activation of cell death regulators (Supplementary Fig. S1A)^6^. Previous studies have demonstrated that inflammatory epithelial cell death and lethality of *Caspase-8* deficient (*Casp8*^ΔIEC^) mice only partially depends on TNF^6^ suggesting that additional factors can trigger cell death. Indeed, recent studies including our own have demonstrated that depending on the cell type and tissue, IFNs can trigger cell death in wildtype and genetically predisposed animals^12–15,17^. Moreover, *Casp8*^ΔIEC^ mice display elevated mRNA copy numbers of several interferons during *Salmonella* infection associated with enhanced STAT1 phosphorylation (Supplementary Fig. S1B–D)^6^. These data strongly suggests a link between IFN-STAT1 signaling and Caspase-8-dependent and -independent cell death during gastrointestinal infection (Supplementary Fig. S1).

**Fig. 1:**
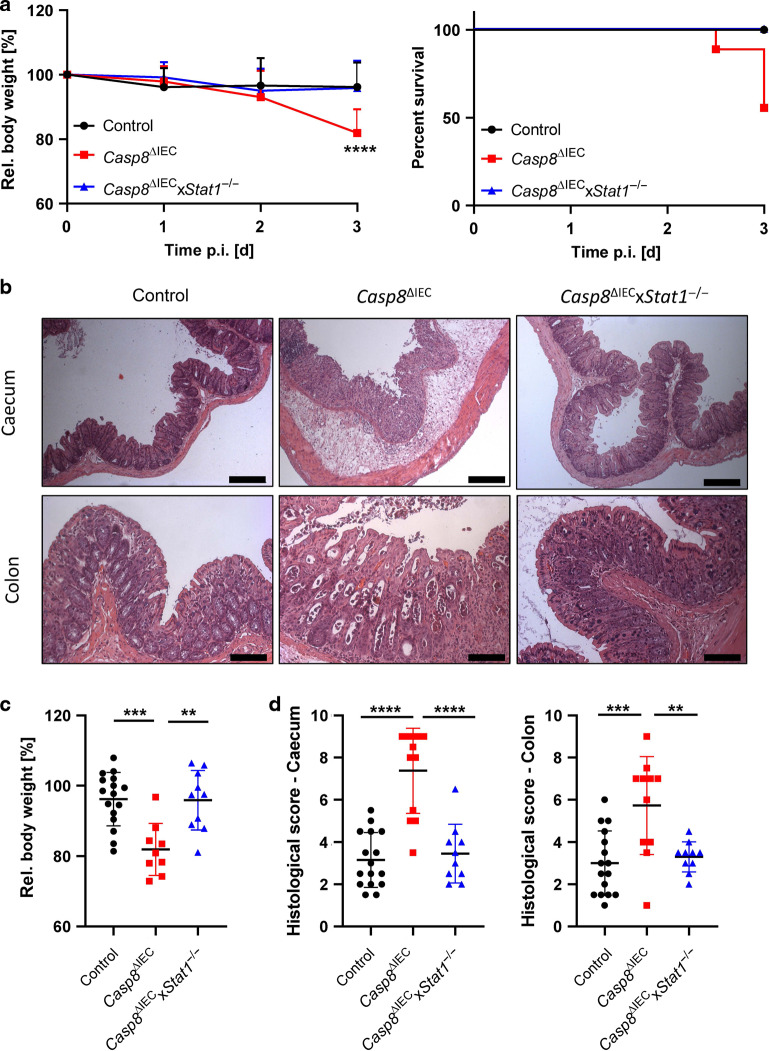
Deletion of STAT1 ensures survival of *Casp8*^ΔIEC^ mice during *Salmonella* Typhimurium infection. **A–D** *Casp8*^ΔIEC,^ *Casp8*^ΔIEC^x*Stat1*^−/−^ mice and control mice were orally infected with *Salmonella* Typhimurium *ΔaroA* and analyzed at day 3 post infection. Pooled data of 4 individual experiments. **A** *Casp8*^ΔIEC^ (*n* = 9), *Casp8*^ΔIEC^x*Stat1*^−/−^ (*n* = 10) mice and control mice (*n* = 16) were orally infected with *Salmonella* Typhimurium *ΔaroA*. Relative body weight and Kaplan–Meier survival curve of infected animals. Error bars indicated +SD. **B** Representative images of caecum and colon cross sections at day 3 post infection with H&E staining (scale bar: caecum 200 μm; colon 100 μm). **C** Relative body weight at day 3 post infection. D Histological scores of H&E stained tissue cross sections. Error bars indicate ±SD. Statistical analyses: ANOVA with Tukey’s multiple comparisons test; NS *p* ≥ 0.05; *p < 0.05; **p < 0.01; ***p < 0.001; ****p < 0.0001.

**Fig. 2:**
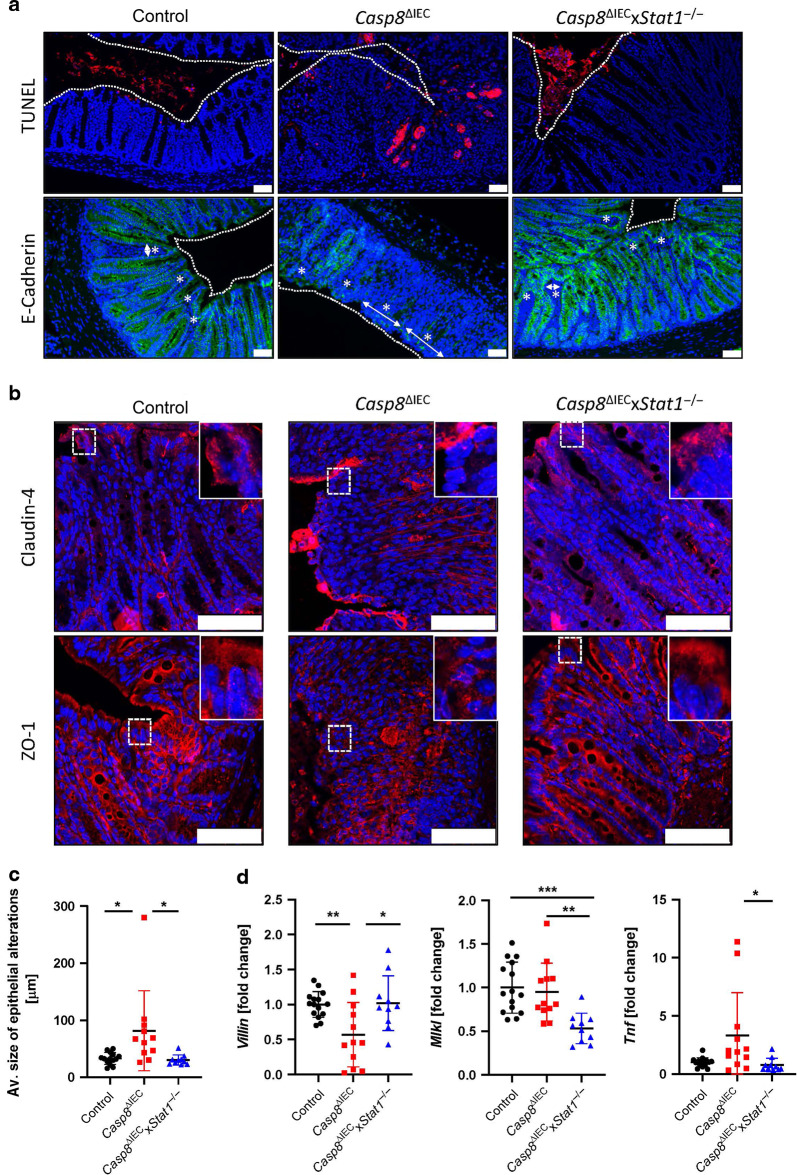
Deletion of STAT1 restores epithelial integrity in *Casp8*^ΔIEC^ mice. **A–D** *Casp8*^ΔIEC^, *Casp8*^ΔIEC^x*Stat1*^−/−^ mice and control mice were orally infected with *Salmonella* Typhimurium *ΔaroA* and analyzed at day 3 post infection. Pooled data of 4 individual experiments. **A** Representative images of colon cross sections immunohistochemically stained with antibody against E-Cadherin (green) or stained with TUNEL assay (red). Nuclei were counterstained with Hoechst 33342 (blue). Tissue gaps are indicated with asterisk and arrows (scale bar: 50 μm). **B** Representative images of colon cross sections immunohistochemically stained with antibody against Claudin-4 or ZO-1 (red) (scale bar: 75 μm). Nuclei were counterstained with Hoechst 33342 (blue). **C** Quantification of epithelial alterations. **D** Gene transcription analysis of colonic mRNA expression. *Hprt* was used as housekeeping gene. Gene expression levels are shown as fold changes. Error bars indicate ±SD Statistical analyses: One-way ANOVA with Tukey’s multiple comparisons test; NS *p* ≥ 0.05; *p < 0.05; **p < 0.01; ***p < 0.001; ****p < 0.0001.

To study the role of STAT1 upstream of Caspase-8 during gastrointestinal infection we subjected control, *Casp8*^ΔIEC^ mice and *Casp8*^ΔIEC^x*Stat1*^−/−^ mice to the streptomycin mouse model for *Salmonella* Typhimurium induced enteritis. As previously described, oral administration of *S*. Typhimurium is associated with infection induced inflammation and tissue destruction starting in the caecum and extending to the colon in wildtype mice^30^. Since *Casp8*^ΔIEC^ mice are highly sensitive to *S*. Typhimurium infection, we used an attenuated *Salmonella* mutant strain (*S*. Typhimurium Δ*aroA*) with reduced virulence. Of note, previous studies have demonstrated that this stain still triggers inflammation and fibrosis in infected mice^31,32^. Remarkably, as previously shown, *Casp8*^ΔIEC^ mice displayed severe tissue damage and dramatic body weight loss associated with high lethality after infection (Fig. 1A, C) due to excessive epithelial cell death resulting in a breakdown of the intestinal barrier (Figs. 1 and 2)^6^. Accordingly, these mice were histomorphologically characterized by severe tissue destruction, accompanied by pronounced edema of the submucosa and inflammatory infiltrates (Fig. 1B, D). Interestingly and in sharp contrast to the phenotype observed in *Casp8*^ΔIEC^ mice, additional deletion of *Stat1* in these mice (*Casp8*^ΔIEC^x*Stat1*^−/−^ mice) could protect from severe endotoxemia, body weight loss and lethality (Fig. 1A, C). This protective effect was further underlined by the fact that double deficient mice displayed minor to no epithelial erosions and epithelial cell death as indicated by H&E staining and quantification of histological tissue damage (Fig. 1B, D). In line with this observation, visualization of cell death by TUNEL (terminal deoxynucleotidyl transferase dUTP nick end labeling) assay as well as characterization of intestinal barrier integrity via E-Cadherin (component of adherens junctions) and tight junction proteins (Claudin-4, Zonula occludens-1), indicated massive necrotic cell death and a heavily impaired barrier function in *Casp8*^ΔIEC^ mice, whereas deletion of *Stat1* restored epithelial integrity and inhibited epithelial cell death (Fig. 2A, B). Control and double deficient mice displayed alterations within the epithelium including intraepithelial vacuoles and cysts but an intact epithelial barrier. These observations suggest an ongoing but controlled infection. In contrast, larger morphological alterations of the epithelium associated with massive tissue destruction that promote epithelial gaps were observed in *Casp8*^ΔIEC^ mice. Epithelial gaps were characterized by a disruption of adherens and tight junctions and a direct exposure of lamina propria cells to the gut lumen (Fig. 2A–C). Accordingly, expression of the intestinal epithelial cell (IEC) marker *Villin* was reduced in *Casp8*^ΔIEC^ mice (Fig. 2D). In line with reduced cell death in *Casp8*^ΔIEC^x*Stat1*^−/−^ mice, increased *Mlkl* gene expression was completely abrogated (Fig. 2D). Additionally, *Tnf* expression, a pro-inflammatory cytokine and well-known driver of cell death was significantly elevated in *Casp8*^ΔIEC^ mice. By sharp contrast *Casp8*^ΔIEC^x*Stat1*^−/−^ mice displayed reduced *Tnf* expression levels (Fig. 2D). In summary, these data demonstrated that STAT1 acts upstream of Caspase-8-independent cell death in the colon during the initial phase of gastrointestinal infection, as lack of this transcription factor in *Casp8*^ΔIEC^ mice was sufficient to block excessive cell death and lethality.

### Epithelial STAT1 is essential to restrict tissue damage during *S*. Typhimurium infection

To further investigate the role of STAT1 during tissue injury response, we analyzed mice 10 days post infection. Interestingly, the protective effect by deletion of *Stat1* in *Casp8*^ΔIEC^mice was stable throughout the course of infection. At day 10 post infection, *Casp8*^ΔIEC^x*Stat1*^−/−^ mice were still alive and had no remarkable changes in bodyweight or displayed histological alterations compared to control mice (Supplementary Fig. S2A, B). Expression levels of *Tnf* as cell death inducer and of *Mlkl* as key factor for necroptosis were significantly higher in control mice (Supplementary Fig. S2C). In line, TUNEL and E-Cadherin staining visualized an intact epithelial barrier with only minor signs of cell death in both strains (Supplementary Fig. S3A), demonstrating that the lack of STAT1 is sufficient to prevent necroptosis. Recruitment of immune cells and particularly macrophages is an essential host defense mechanism during infections to restrict and eliminate pathogens^3,4^. We could observe only minor differences in the numbers of mucosal macrophages or neutrophil granulocytes (Supplementary Fig. S3B) or in mRNA copy numbers of inflammatory markers like *S100a9* and *Reg3g* (Supplementary Fig. S2C) during the course of infection between control and double deficient mice. In summary, these data suggest that STAT1 has a key function in the intestinal epithelium by orchestrating cell death as a host response during intestinal infection with *S*. Typhimurium.

### STAT1 maintains tissue homeostasis during *Citrobacter rodentium* infection

Having shown that STAT1 is involved in orchestrating Caspase-8-independent programmed necrosis during infection with the intracellular pathogen *Salmonella*, we were interested whether this is a widely used host defense mechanism during gastrointestinal infection. To address this, we took advantage of the *C. rodentium* model, a murine pathogen that mimics human infections with attaching and effacing *Escherichia coli* (EHEC/EPEC). While a previous study has demonstrated an essential role of the IL-22-STAT3 signaling cascade during *C. rodentium* infection, the role of STAT1 has not been elucidated^33^. To provide functional evidence for a role of STAT1 in controlling Caspase-8-dependent and -independent cell death during *C. rodentium* infection, we infected control, *Casp8*^ΔIEC^ mice and *Casp8*^ΔIEC^x*Stat1*^−/−^ mice.

**Fig. 3:**
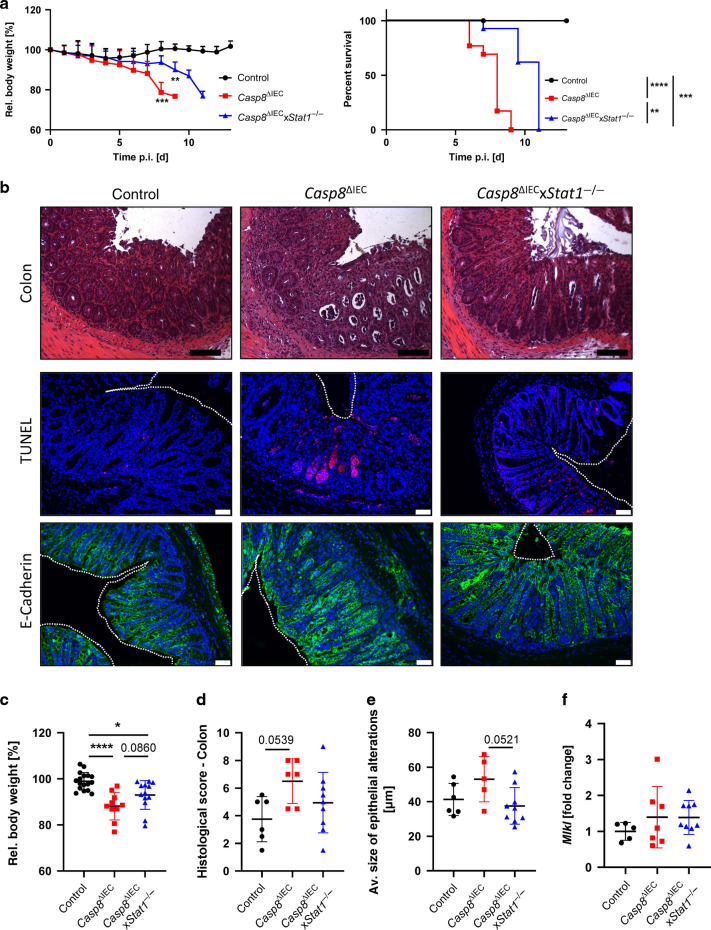
STAT1 contributes to epithelial cell death during *Citrobacter rodentium* infection. **A**-F *Casp8*^ΔIEC^, *Casp8*^ΔIEC^x*Stat1*^−/−^ mice and control animals were orally infected with *Citrobacter rodentium* and analyzed at day 7 post infection. Pooled data of individual experiments (**A**, **C** *n* = 4; **B**, **D**–**F** *n* = 2). **A** *Casp8*^ΔIEC^ (*n* = 13), *Casp8*^ΔIEC^x*Stat1*^−/−^ (*n* = 13) mice and control mice (*n* = 16) were orally infected with *Citrobacter rodentium*. Relative body weight and Kaplan–Meier survival curve of infected animals. Error bars indicated +SD. **B** Representative images of H&E stained colon cross sections (scale bar: 100 μm) or immunohistochemically stained with an antibody against E-Cadherin (green) or stained with TUNEL assay (red). Nuclei were counterstained with Hoechst 33342 (blue) (scale bar: 50 μm). **C** Rel. bodyweight at day 7 post infection. **D** Histological score of H&E stained tissue cross sections. **E** Quantification of epithelial alteration of E-Cadherin staining visualized in **B**. **F** Gene transcription analysis of colonic mRNA expression. *Hprt* was used as housekeeping gene. Gene expression level is shown as fold change. Error bars indicate ±SD. Statistical analyses: One-way AOVA with Tukey’s multiple comparisons test; NS *p* ≥ 0.05; *p < 0.05; **p < 0.01; ***p < 0.001; ****p < 0.0001.

Interestingly, *C. rodentium* infection leads to a similar lethal disease outcome in mice lacking C*aspase-8* in the intestinal epithelium as observed during *Salmonella* infection (Fig. 3). Accordingly, Casp8^ΔIEC^ mice displayed a dramatic weight loss starting from day 5 post infection and all mice died within 10 days (Fig. 3A, C). Similar to *Salmonella* infection, massive epithelial cell death, associated with enormous necrotic areas was accompanied by severe tissue destruction and loss of epithelial integrity (Fig. 3B, D). Interestingly, while previous studies suggested a major role for STAT3 during *Citrobacter* infection, our data suggest that STAT1 partially contributes to the host defense of extracellular pathogens. Accordingly, the bodyweight of control, *Casp8*^*ΔIEC*^ and *Casp8*^*ΔIEC*^*xStat1*^*−/−*^ mice remained similar up to day 5, but after this time point *Casp8*^*ΔIEC*^ mice show a rapid weight loss and high lethality at day 9, whereas the majority of *Casp8*^*ΔIEC*^*xStat1*^*−/−*^ mice were still alive with only a slight weight loss (Fig. 3A). At day 7, when we sacrificed the mice for detailed analyzes, *Casp8*^*ΔIEC*^*xStat1*^*−/–*^ mice displayed still a moderate weight loss (Fig. 3C). Moreover, at a histological level *C. rodentium* infection associated cell death and barrier dysfunction were clearly evident in *Casp8*^*ΔIEC*^ mice as demonstrated by a dramatic loss of crypt-villus architecture, an enormous number of infiltrating immune cells and massive cell death. In sharp contrast, control and *Casp8*^*ΔIEC*^*xStat1*^*−/−*^ mice exhibited the previously published hyperplasia^34^ with comparable levels of cell death and barrier integrity (Fig. 3B). The central contribution of STAT1 upstream of Caspase-8 and necroptosis in the context of *C. rodentium* infection was further supported in the histological score and quantification of alterations in the intestinal tissue (Fig. 3D, E). Interestingly, we could not observe differences in *Mlkl* mRNA expression between *Casp8*^*ΔIEC*^ mice and *Casp8*^*ΔIEC*^*xStat1*^*−/−*^ animals (Fig. 3F).

### STAT activation during gastrointestinal infection

**Fig. 4:**
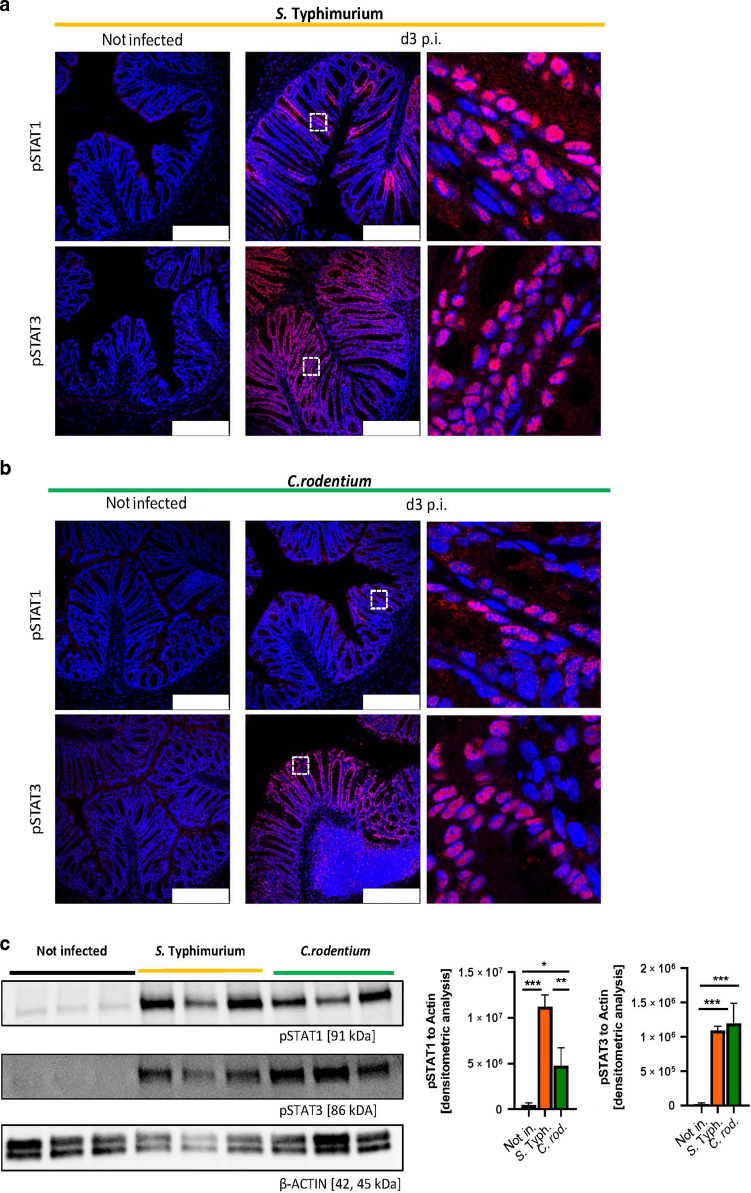
STAT1 and STAT3 activation during *Salmonella* Typhimurium and *Citrobacter rodentium* infection. Control animals (n ≥ 6) were infected with *S*. Typhimurium *ΔaroA* (**A**) or *C. rodentium* (**B**) sacrificed at day 3 post infection. Representative images of colon cross sections immunohistochemically stained with antibody against pSTAT1 (scale bar: 250 μm) or pSTAT3 (scale bar: 250 μm) (red). Nuclei were counterstained with Hoechst 33342 (blue). **C** Western Blot analysis and normalization of colonic tissue with antibodies against pSTAT1 and pSTAT3. β-Actin was used as loading control. Densitometry analysis for quantification (*n* = 3 per group). Error bars indicate +SD. Statistical analyses: One-way ANOVA with Tukey’s multiple comparisons test; NS *p* ≥ 0.05; *p < 0.05; **p < 0.01; ***p < 0.001.

STAT3 is predominantly known to mediate epithelial recovery and tissue regeneration^35^, whereas STAT1 has been described as modulator of cell death^12,13^. To better delineate the contribution of the IFN-STAT1 and the IL-22-STAT3 axis in the context of gastrointestinal infection, we compared phosphorylation of STATs in the two different infection models. STAT3 was activated at high levels in both types of infection along the entire crypt-villus axis, as displayed in the broad signal of phosphorylated STAT3 (pSTAT3 at Tyr705) in immunohistochemical staining (Figs. 4A, B) and in the quantification of STAT3 phosphorylation by Western Blot (pSTAT3 at Tyr705 Fig. 4C, pSTAT3 at Ser727 Supplementary Fig. S4). In contrast, activation of STAT1 by phosphorylation at Tyr701 was restricted to defined areas and was most prominent in epithelial cells at the surface epithelium, which is exposed to luminal contents including invading and attaching pathogens (Fig. 4B). In the context of invading pathogen (*S*. Typhimurium), phosphorylated STAT1 was detectable in epithelial cells at the surface epithelium and deep in the crypt (Fig. 4A). In sharp contrast, during *Citrobacter* infection, STAT1 activation was limited to highly defined areas within the crypt region. Western Blot analysis confirmed an increased STAT1 phosphorylation at Tyr701 during *Salmonella* induced enteritis compared to tissue derived from *C. rodentium* infection and steady state conditions (Fig. 4C, Supplementary Fig. S4). In conclusion, these results indicate that STAT1 signaling is activated during both types of infection and most prominent at the villus epithelium, suggesting that STAT1 signaling is involved in cell extrusion during bacterial infection by controlling epithelial cell death, while STAT3 has an important role in tissue injury responses following STAT1 mediated cell loss.

### STAT1 transcriptionally controls central cell death pathways in the intestinal epithelium during *Salmonella* infection

**Fig. 5:**
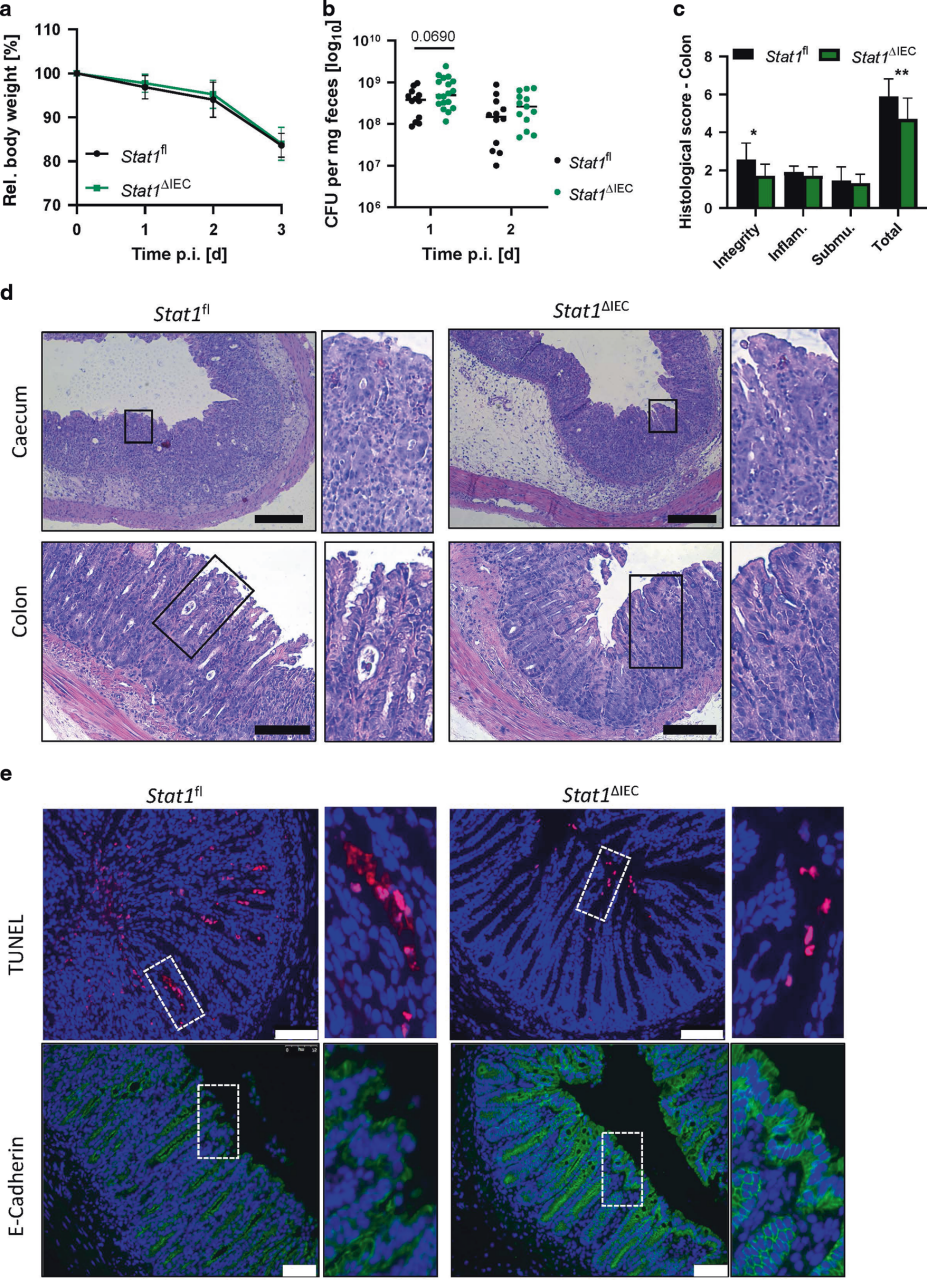
Epithelial STAT1 signaling coordinates the host defense during *Salmonella* Typhimurium infection. **A–E** *Stat1*^*ΔIEC*^ mice and control littermates were orally infected with *Salmonella* Typhimurium (SL1344) and analyzed at day3 post infection. Pooled data of independent individual experiments (*n* ≥ 4). **A** *Stat1*^*ΔIEC*^ mice (*n* = 11) and control (*Stat1*^*fl*^) littermates (*n* = 14) were orally infected with *Salmonella* Typhimurium. Relative body weight of infected animals Error bars indicated ± SD. **B** Bacterial burden of the first two days post infection. **C** Histological scores of H&E stained colonic tissue cross sections. Error bar + SD. NS *p* ≥ 0.05; *p < 0.05; **p < 0.01. **D** Representative images of caecum and colon cross sections with H&E staining (scale bar: caecum 200 μm; colon 100 μm). **E** Representative images of colon cross sections immunohistochemically stained with antibody against E-Cadherin (green; scale bar: 75 μm), or stained with TUNEL assay (red; scale bar: 100 μm). Nuclei were counterstained with Hoechst 33342 (blue).

**Fig. 6:**
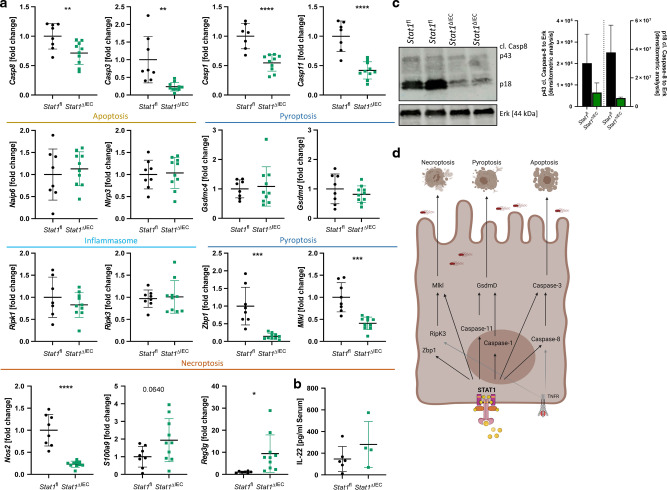
Intestinal epithelial STAT1 signaling coordinate host cell death during wildtype *Salmonella* Typhimurium infection. **A–C** *Stat1*^*ΔIEC*^ mice and control (*Stat1*^*fl*^) littermates were orally infected with *Salmonella* Typhimurium (SL1344) and analyzed at day3 post infection. Experiments were repeated 3 times with similar results (pooled data). **A** Gene transcription analysis of colonic mRNA expression. *Gapdh* was used as housekeeping gene. Gene expression level is shown as fold change. Error bars indicate ±SD. Statistical analyses: Student’s *t* test; NS *p* ≥ 0.05; *p < 0.05; **p < 0.01; ***p < 0.001; ****p < 0.0001. **B** IL-22 serum ELISA. Error bars indicate ±SD. **C** Western blot analysis and normalization of colonic tissue with antibodies against cleaved Caspase-8. Erk was used as loading control. Densitometry analysis for quantification. **D** Schematic overview of cell death pathways altered by STAT1. Created with www.BioRender.com.

To determine whether epithelial STAT1 signaling participates in the host response against *S*. Typhimurium by orchestrating epithelial cell death particularly as early defense, we infected mice lacking *Stat1* specifically in the intestinal epithelium (*Stat1*^ΔIEC^). Similar to control mice, *Stat1*^ΔIEC^ mice lost body weight following oral infection with wildtype *S*. Typhimurium (Fig. 5A). While we could not observe differences in body weight loss, we discovered an elevated bacterial burden in the feces of *Stat1*^ΔIEC^ mice, particularly at day one post infection (Fig. 5B). When looking at barrier function and cell shedding, we observed that the lack of epithelial STAT1 strongly diminished extrusion of epithelial cells both in the caecum and in the colon (Fig. 5D, E). Histomorphological analyses uncovered that barrier integrity was more preserved in *Stat1*^ΔIEC^ mice (lower histological score), whereas features such as pathophysiological alterations of the submucosa including edema were similar between deficient and control mice (Fig. 5C, D). Accordingly, we could not observe alterations in the number of invading immune cells at this early time point during infection (Supplementary Fig. S5A). However, in line with the enhanced barrier integrity, the numbers of TUNEL positive cells in the lumen and epithelium were strongly reduced in *Stat1*^ΔIEC^ mice. While E-Cadherin staining revealed weakening of the epithelial barrier with fragile adherent junctions in control mice, deficient animals showed intact epithelial lining (Fig. 5D, E). Interestingly, TNF serum levels were comparable between both groups (Supplementary Fig. S5B), demonstrating that cell death frequency is not caused by diminished presence of cell death activators. To further delineate the impact of STAT1 on cell extrusion in the epithelium, we evaluated mRNA expression of a broad range of key members of central cell death pathways. Interestingly, we observed that the lack of the transcription factor STAT1 affected gene expression of several cell death pathways (Fig. 6A). Accordingly, gene transcription of central molecules of three of the most prominent cell death pathways associated with *S*. Typhimurium infection, apoptosis, necroptosis and pyroptosis, were all strongly diminished in *Stat1*^ΔIEC^mice. The key caspase for extrinsic apoptosis including the initiator *Caspase-8* and the effector *Caspase-3*, as well as *Caspase-1* and *Caspase-11* involved in pyroptosis were significantly downregulated. Additionally, Western Blot analyses revealed that STAT1 deficient mice displayed a strongly reduced activation of the central cell death regulator Caspase-8. Importantly, we did not only observe a reduction in the p18 subunit (fully activated form), but also a dramatic reduction of the partially activated form (p43), which is important to block necroptosis (Fig. 6C). Furthermore, gene transcription of *Mlkl*, as well as of *Zbp1*, recently described as upstream regulator of necroptosis^11^, were dramatically impaired. In sharp contrast, *Ripk1* expression as key switch between apoptosis and necroptosis^11^ was not influenced by the deletion of STAT1. Moreover, members of the inflammasome (*Naip6*, *Nlrp3*) and associated molecules involved in pyroptosis downstream of Caspase-1 and Caspase-11 (*Gsdmc*, *Gsdmd*) were not altered (Fig. 6A). We further observed a dramatic reduction of the NF-κB target gene *iNos* in mice lacking STAT1 in the intestinal epithelium (Fig. 6A), suggesting a role of STAT1 upstream of this signaling pathway that has to be further evaluated^36^. Moreover, as elimination of infected cells is essential during host defense against invading pathogens, *Stat1*^ΔIEC^mice displayed elevated levels of the pro-inflammatory marker *S100a9* and IL-22 serum levels as well as enhanced transcription of the antimicrobial peptide *Reg3g* (Fig. 6A, B).

In summary, our results suggest an important role of epithelial STAT1 during gastrointestinal infection by coordinating casaspase-8-dependent and -independent cell death pathways (Fig. 6D).

## Discussion

Cell extrusion is an essential host defense mechanism during gastrointestinal infection to limit pathogen tissue loads, to hamper pathogen spread and to eliminate the invading pathogen. Accordingly, infection of the gastrointestinal tract by bacterial or viral pathogens is associated with epithelial cell death during the initial phase of infection and often accompanied by tissue injury responses involving epithelial renewal^37,38^. Cell death in the intestinal epithelium involves multiple signaling pathways that are activated depending on the physiological/pathophysiological context, and precise mechanistic knowledge of the cell death machinery and the essential upstream components in gastrointestinal infection relevant for therapeutic aspects is limited so far. In this study, we uncovered that epithelial STAT1 signaling orchestrates Caspase-8-dependent and -independent epithelial cell death during gastrointestinal infection. On a molecular level, we unravel that activation of STAT1 in the intestinal epithelium was linked to gene expression of molecules involved in apoptosis (*Caspase-8*, *Caspase-3*), necroptosis (*Mlkl*, *Zbp1*) as well as pyroptosis (*Caspase-1*, *Caspase-11)* (Fig. 6), suggesting that transcriptional control of STAT1 at the crossroad of several essential cell death pathways is an essential host response mechanism, required for the maintenance of the intestinal barrier. Our data further suggest that this pathway has to be tightly controlled, as genetic deletion of one of these pathways was associated with systemic infection and lethality. Accordingly, dysregulation of Caspase-8, as a central regulator for apoptosis and necroptosis was associated with excessive STAT1 mediated cell death accompanied by systemic bacterial spreading (Fig. 1). By contrast, modulation of several cell death pathways via STAT1 deletion (Fig. 5) restricts cell extrusion and allowed pathogen to colonize the host. Hence strict controlled and coordinated cell death is required to ensure host survival^4–6,14–16,39^.

Previous publications highlighted the role of pyroptosis for cell death and cell extrusion during the course of infection including early and late stages of infection. Within the first 36 h after Salmonella infection the Naip/NLRC4 inflammasome is required for Caspase-1 mediated cell extrusion associated with IL-18 production whereas at a later time point (>72 h post infection) non-canonical inflammasome activation involving Caspase-11 is needed^7,40^. Caspase-1 seems to be essential at baseline to provide pro-inflammatory signaling required for Caspase-11 function and both, canonical and non-canonical inflammasome activation, have their distinct impact on pathogen restriction and host defense^41^. Later studies uncovered that cell extrusion can occur in a lytic or apoptotic form but also in a mixed phenotype involving mechanisms controlled by lytic and apoptotic caspases^42^. Man et al. as well as Rauch et al. described a contribution of Caspase-8 to NLRC4 inflammasome and associated IL-1β and IL-18 production^8,43^. In addition, we previously published that during the early phase of oral *S*. Typhimurium infection (ca. 72 h post infection), apoptotic caspases as well as mediators for necroptosis, as a novel form of lytic cell death, are activated in wildtype mice^6^. While it is commonly accepted that apoptosis and pyroptosis are the main mechanisms of cell death in response to infection, current studies are increasingly focusing on the role of necroptosis^4,44^. Necroptosis was already described for macrophages during *Salmonella* infection induced by type I interferons, associated with immune escape and bacterial spread^15,16,34^. Accordingly, macrophages deficient in IFNαR1 were highly resistant to *Salmonella* mediated necroptosis associated with enhanced pathogen control^16^. In line with this, here we uncovered that STAT1 is essential to coordinate necroptosis in intestinal epithelial cells during *Salmonella* and *Citrobacter rodentium* infection. Dysregulated control of cell death (*Casp8*^ΔIEC^ mice) was associated with bacterial spread and reduced survival. Loss of Caspase-8 as central cell death regulator enables massive necroptotic cell death, which was only partial dependent on TNF^6^. In our current study, we delineated that this harmful pathway is controlled by STAT1, as deletion of this transcription factor was sufficient to block excessive cell death in *Casp8*^ΔIEC^ (Figs. 1, 2, and 3).

Our results derived from the infection of *Stat1*^ΔIEC^ animals uncovered the manifold role of intestinal STAT1 signaling for the control of Caspase-dependent and -independent cell death (Fig. 6). Interestingly, a recent publication reported a flexible usage and interconnectivity of several cell death pathways including pyroptosis and apoptosis occurring in macrophages required to control and eliminate intracellular pathogens^39^. In line with this, our study demonstrates that key elements of these cell death pathways are regulated via IFNs and STAT1, uncovering STAT1 as a key factor at the crossroad of these cell death pathways. In line with this, previous publications linked interferons to the control of bacterial infection by activating pyroptosis^41,44,45^ as well as to the amplification of inflammation by transcriptional control of necroptosis and apoptosis^12,13,46,47^.

Interestingly, STAT1 mediated cell elimination seems to be a widely used host defense mechanism as we observed activation of STAT1 in the surface epithelium of mice infected with *S*. Typhimurium and *C. rodentium*. Of note, phosphorylation of STAT1 was more pronounced during *S*. Typhimurium infection, while activation of STAT3 was similar between both models (Fig. 4, Supplementary Fig. S4), suggesting that in the context of *C. rodentium* further cell death mechanisms independent of STAT1 tyrosine phosphorylation might be involved. Of note, in the context of *C. rodentium* infection, STAT3 was described as an essential player for the intestinal epithelium to restrict infection by suppressing cell death to maintain intestinal integrity, which might be an alternative hypothesis for reduced STAT1 phosphorylation^33^. Furthermore, STAT signaling in combination with interleukins are a complex signaling network with manifold settings and feedback loops as well as backup systems. In this context, upregulation of *S100a9* and *Reg3g* as well as elevated IL-22 serum levels during *Salmonella* infection (Fig. 6) suggesting alternative signaling pathways potentially mediated via STAT3^48^. Interestingly, previous studies have demonstrated that IL-22 signaling, alone or in combination with STAT3, is essential for maintaining intestinal epithelial integrity during *Salmonella* infection^49–51^. In summary, multiple pieces of evidence demonstrate that STAT1 and STAT3 both have essential functions during gastrointestinal infection by orchestrating epithelial barrier integrity. However further studies are required to delineate if and how the IFN-STAT1 and IL-22-STAT3 axis interact to control bacterial infection.

Furthermore, our data underline the oppositional function of STAT1 for cell death regulation during bacterial infection. Whereas deletion of STAT1 in the context of massive necroptosis improves host survival (Fig. 1), STAT1 deletion in mice that are not genetically predisposed to inflammatory cell death hampers the extrusion of infected cells. However, this is an important host mechanism to clear infection (Fig. 5). In line with this, loss- as well as gain-of function mutations of *STAT1* in humans are associated with susceptibility to infections^24–29^. Increased STAT1 activation as well as diminished activation can both have negative effects for the host depending on the cell type, tissue and disease context. Interestingly, in the context of gastrointestinal inflammation, such as inflammatory bowel disease, STAT contributes to cell death in the small intestine, while in the context of colitis it controls mucosal healing downstream of IFN-λ^13,52,53^. Within this manuscript, we now demonstrate that in the context of gastrointestinal infection, STAT1 mediates epithelial cell death in the colon that might be an essential step to prevent infection, but is detrimental in genetically predisposed mice. Accordingly, additional deletion of STAT1 in *Casp8*^ΔIEC^ mice can rescue lethality during *Salmonella* induced enteritis (Fig. 1), ameliorates *C. rodentium* infection (Fig. 3) but is not sufficient to prevent tissue destruction during DSS colitis^53^ highlighting the context-specific functions of STAT1. Moreover, a dual function of STAT1 signaling is also highly discussed during intestinal tumor formation including cell death regulation and immune cell modulation^54–56^.

In summary, our study reveals that interferons and STAT1 signaling play a crucial role during early host defense against bacterial pathogens by regulating a complex cell death network. Dysregulation of this network by lacking central regulators can cause either massive cell death supporting inflammation and bacterial spread or alternatively prevent cell death diminishing bacterial elimination. We therefore propose that STAT1 functions at a convergent point of multiple cell death mechanisms that is essential for host survival and the restriction of bacterial growth.

## Material and methods

### Mice

*Casp8*^ΔIEC^^57^, *Stat1*^−/−^^58^, *Stat1*^ΔIEC^^52^ mice were described earlier. *Casp8*^ΔIEC^x*Stat1*^−/−^ were generated by crossing *Casp8*^ΔIEC^ mice to *Stat1*^−/−^ mice. As controls we used littermates (*Casp8*^fl^ or *Stat1*^fl^) or C57BL/6 mice. For all experiments, female and male mice between 6 and 12 weeks were used. All mice were housed in individually ventilated cages. At the end of the experiments, mice were sacrificed by cervical dislocation. Mice were routinely screened for pathogens according to FELASA guidelines. Animal protocols were approved by the Institutional Animal Care and Use Committee of the Regierung von Unterfranken.

### *Salmonella* Typhimurium infection

The *Salmonella enterica* serovar Typhimurium strains Δ*aroA*^31^ and a wild type strain (SL1344) were cultured at 37 °C in sterile LB-media, supplemented with streptomycin, under continuous shaking and aeration and resuspended in sterile PBS for infection. For in vivo infection, mice were treated with a single dose of streptomycin by oral gavage 24 h before infection like previously described^25^. Prior to infection, mice were starved for 6–8 h and subsequently orally gavaged with 10^7^–10^8^ colony forming units (CFU) of *Salmonella* (Δ*aroA* or wild type). For quantification of the applied numbers of *Salmonella*, the bacterial suspension was plated on agar plates.

### Citrobacter rodentium infection

The luminescent strain of *Citrobacter rodentium* (C. rodentium strain ICC169)^59^ was cultured at 37 °C in sterile LB-media, supplemented with erythromycin, under continuous shaking and aeration and resuspended in sterile PBS for infection. Before infection, mice were starved for 6–8 h and subsequently orally gavaged with ~10^9^ colony forming units of *Citrobacter*. For quantification of the applied numbers of *Salmonella*, the bacterial suspension was plated on agar plates.

### Organ collection and storage

Tissue for histology and immunohistochemistry were collected and fixed in 4.5% formaldehyde. Tissue was embedded in paraffin in a water-free procedure and stored at room temperature for further analysis. Samples for cryo-sections, RNA and protein analyses were instantly frozen in liquid nitrogen and stored at −80 °C until further use.

### Bacterial enumeration

Feces was collected in sterile phosphate-buffered saline (PBS) and homogenized. Serial dilutions of the homogenates were plated on LB-Agar plates with streptomycin and incubated at 37 °C overnight.

### Histology and immunohistochemistry

For immunohistochemical analysis, frozen tissue slices were fixed on glass slides using 4% PFA and paraffin-embedded tissue sections were dewaxed and rehydrated. Histopathological analyses were performed on formalin-fixed paraffin-embedded tissue cross sections after Mayer’s haematoxylin and eosin (H&E) staining. Immunofluorescence of tissue sections was performed using a biotinylated secondary antibody together with the TSA Cy3/Fluorescein system as recommended by the manufacturer (Perkin Elmer) or with Streptavidin Protein DyLight (Thermo). Primary antibodies (for detailed information see Supplementary Table 1) were incubated overnight. Nuclei were counterstained with Hoechst 33342 (Invitrogen). Cell death (TUNEL) was analyzed using the In-Situ Cell Death Detection Kit (Roche). Staining for immune cells was performed on cryo-sections or paraffin sections. Images were obtained using a confocal fluorescence microscope LEICA TCS SP5 II, the microscope LEICA DMI 4000B together with the LEICA DFC360 FX or LEICA DFC420 C camera or the microscope Leica DMi1 with the corresponding imaging software. Quantification of epithelial alterations by measurement of size were adapted as previously published by Fattinger et al.^60^. Epithelial alteration includes intraepithelial vacuoles, cysts and epithelial gaps.

### Pathology scoring

Pathology scoring was performed on H&E stained tissue sections by averaging the total amount of (a) Integrity of the intestinal epithelium(0–3) (b) Mucosal inflammation(0–3) and (c) Submucosa(0–3) according our precious publication^7^.

### Gene expression analysis

Total RNA was extracted from whole intestinal tissue using the peqGOLD Total RNA Kit (peqLab/VWR). cDNA was synthesized by reverse transcription using the SCRIPT cDNA Synthesis Kit (Jena Bioscience) and analyzed by real-time qPCR using SYBRGreen reagent (Roche), the LightCycler 480 (Roche) and specific QuantiTect Primer Assays (Qiagen) or primers ordered from Biomers (for detailed information see Supplementary Table 2). PCR product specificity was verified by performance of a melting curve for each primer set. Experimental values were normalized to levels of the housekeeping gene *hypoxanthine guanine phosphoribosyl transferase* (*Hprt*) or *Glyceraldehyde 3-phosphate dehydrogenase* (*Gapdh*). For fold change calculation, the average mean of the relative expression of control mice were set as 1.

### Immunoblotting

Proteins were isolated from whole colon tissue using cell lysis buffer (Cell Signaling) supplemented with PMSF, PhosSTOP (Roche) and cOmplete Protease-Inhibitor (Roche). Lysates were centrifuged and proteins were separated using a MiniProtean-TGX stain free gel (Bio-Rad) and transferred to a nitrocellulose membrane (Bio-Rad). Membranes were blocked (BSA or milk) and probed with primary antibodies (for detailed information see Supplementary Table 1). HRP-linked anti-rabbit was used as a secondary antibody. Blots were developed by chemiluminescence using an ECL substrate (Perkin Elmer). Chemiluminescent signal was detected with a ChemiDoc (Biorad) or Amersham ImageQuant 800 (Cytiva). Western Blot quantification was performed with ImageLab (Biorad).

### ELISA

ELISAs to determine serum levels of IL-22 (R&D) and TNF (R&D) were performed according the user manual.

### Statistical analyses

Comparisons of two groups were performed using an unpaired two-tailed Student’s *t* test. Comparisons among multiple groups were performed using ANOVA followed by multiple comparison and statistical significance was accepted with *p* < 0.05 (NS *p* ≥ 0.05; *p < 0.05; **p < 0.01; ***p < 0.001; ****p < 0.0001). Kaplan–Meier survival curve and associated statistical analysis as well as statistical calculations were performed using GraphPad Prism 8 (GraphPad Software).

### Graphical summary

Graphics were created with www.BioRender.com.

## Supplementary information

Supplementary Material
