# Supplementary material for: STAT1 coordinates intestinal epithelial cell death during gastrointestinal infection upstream of Caspase-8
Source: Mucosal Immunol. 2021 Sep 8;15(1):130–42. doi: 10.1038/s41385-021-00450-2 (PMC8732278; doi:10.1038/s41385-021-00450-2)
Supplement: Supplementary file 1 — Supplementary Material [file 41385_2021_450_MOESM1_ESM.pdf]

Supplementary

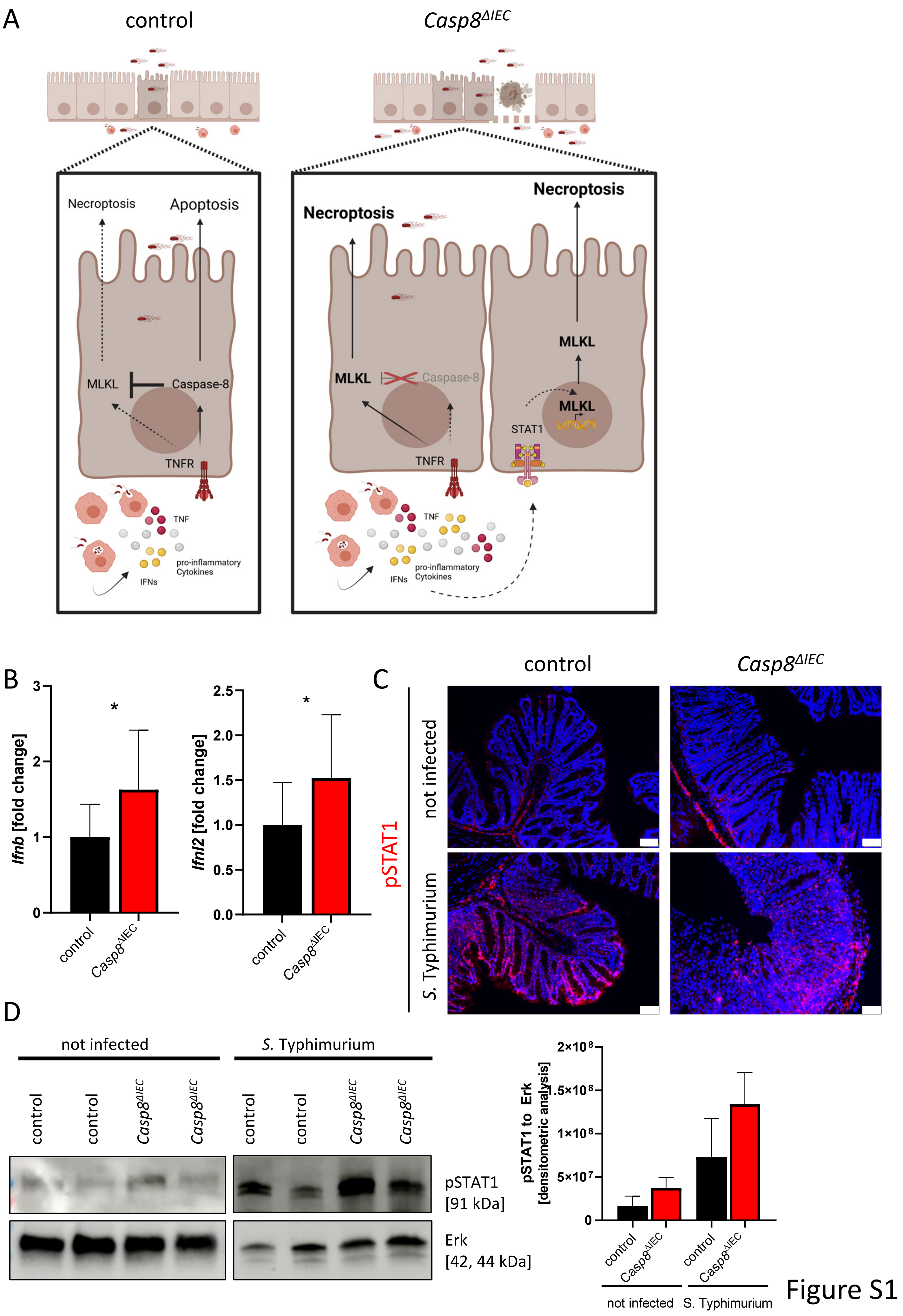
A
control
Casp8ΔIEC
Necroptosis
Apoptosis
MLKL
Caspase-8
TNFR
TNF
pro-inflammatory Cytokines
IFNs
Necroptosis
Necroptosis
MLKL
Caspase-8
MLKL
MLKL
STAT1
TNFR
TNF
pro-inflammatory Cytokines
IFNs
B
Ifnb [fold change]
Ifnl2 [fold change]
control
Casp8ΔIEC
C
control
Casp8ΔIEC
not infected
S. Typhimurium
pSTAT1
D
not infected
S. Typhimurium
control
control
Casp8ΔIEC
Casp8ΔIEC
control
control
Casp8ΔIEC
Casp8ΔIEC
pSTAT1 [91 kDa]
Erk [42, 44 kDa]
pSTAT1 to Erk [densitometric analysis]
control
Casp8ΔIEC
control
Casp8ΔIEC
not infected
S. Typhimurium

Figure S1

Figure S1: STAT1 activation during *Salmonella* infection

A) Schematic overview on the role of Caspase-8 for epithelial barrier function during *Salmonella* Typhimurium infection. In wildtype mice (left) pathogen invasion is restricted due to the elimination of infected cells by apoptosis. TNF can trigger Caspase-8-dependent (apoptosis) or -independent cell death (necroptosis). During gastrointestinal infection, intestinal epithelial cells or mucosal immune cells release several cytokines to promote host defense. In the absence of Caspase-8 (right), necroptosis disrupts the epithelial barrier, enables bacterial invasion and systemic spread and increases the release of pro-inflammatory cytokines. In this context, interferons are strongly connected to an induction of MLKL expression via activation of the transcription factor STAT1. Created with BioRender.com. **B-D)** *Casp8*$^{\Delta IEC}$ and control mice were left untreated or orally infected with *Salmonella* Typhimurium *ΔaroA* and analyzed at day 3 post infection. **B)** Gene transcription analysis of colonic mRNA expression. *Hprt* was used as housekeeping gene. Gene expression levels are shown as fold changes. Statistical analyses: Student's t-test; NS $p \geq 0.05$; *** $p < 0.001$. Pooled data of 3 independent experiments. **C)** Representative images of colon cross sections immunohistochemically stained with antibody against pSTAT1 (Tyr701) (red). Nuclei were counterstained with Hoechst 33342 (blue). **D)** Western Blot analysis and normalization of colonic tissue with antibody against pSTAT1. Erk was used as loading control. Densitometry analysis for quantification (average of n=2 per group).

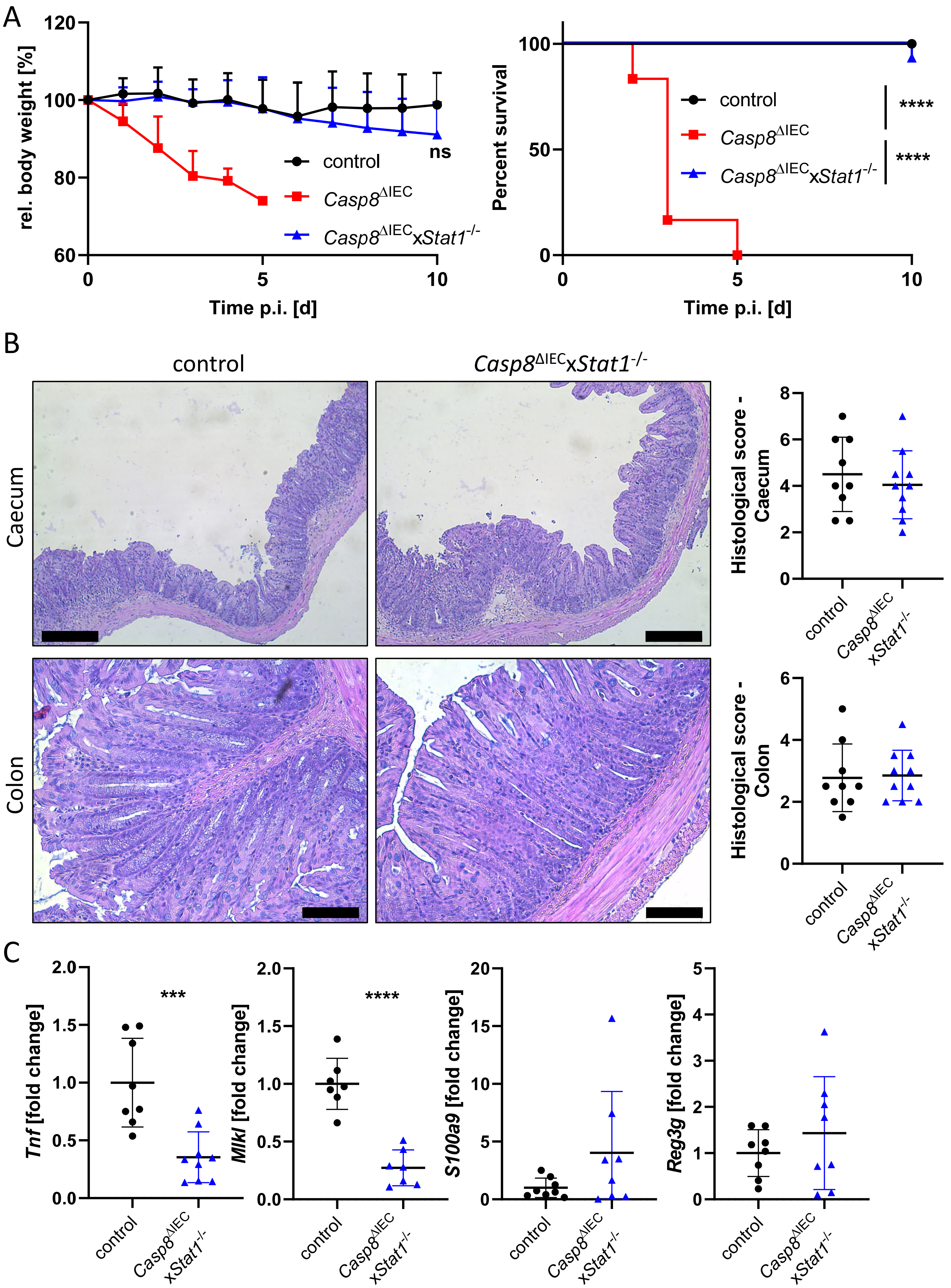
A
rel. body weight [%]
Time p.i. [d]
control
Casp8ΔIEC
Casp8ΔIECxStat1-/-
ns
Percent survival
****
****
B
control
Casp8ΔIECxStat1-/-
Caecum
Colon
Histological score - Caecum
Histological score - Colon
C
Tnf [fold change]
Mlkl [fold change]
S100a9 [fold change]
Reg3g [fold change]
***
****

Figure S2

Figure S2: Role of STAT1 during late phase of infection

A-D) *Casp8*$^{\Delta IEC}$x*Stat1*$^{-/-}$ mice and control mice were orally infected with *Salmonella* Typhimurium *ΔaroA* and analyzed at day 10 post infection. Pooled data of 3 individual experiments. **A)** *Casp8*$^{\Delta IEC}$ (n=6), *Casp8*$^{\Delta IEC}$x*Stat1*$^{-/-}$ (n=14) mice and control mice (n=11) were orally infected with *Salmonella* Typhimurium *ΔaroA*. Relative body weight and Kaplan-Meier survival curve of infected animals. Error bars indicated + SD. **B)** Representative images of caecum and colon cross sections at day 10 post infection with H&E staining (scale bar: caecum 200 µm; colon 100 µm). Corresponding histological scores of H&E stained tissue cross sections. **C)** Gene transcription analysis of colonic mRNA expression. *Gapdh* was used as housekeeping gene. Gene expression levels are shown as fold changes. Each point represents one mouse. Error bars indicate +/- SD. Statistical analyses: Student's t-test; NS $p \geq 0.05$; * $p < 0.05$.

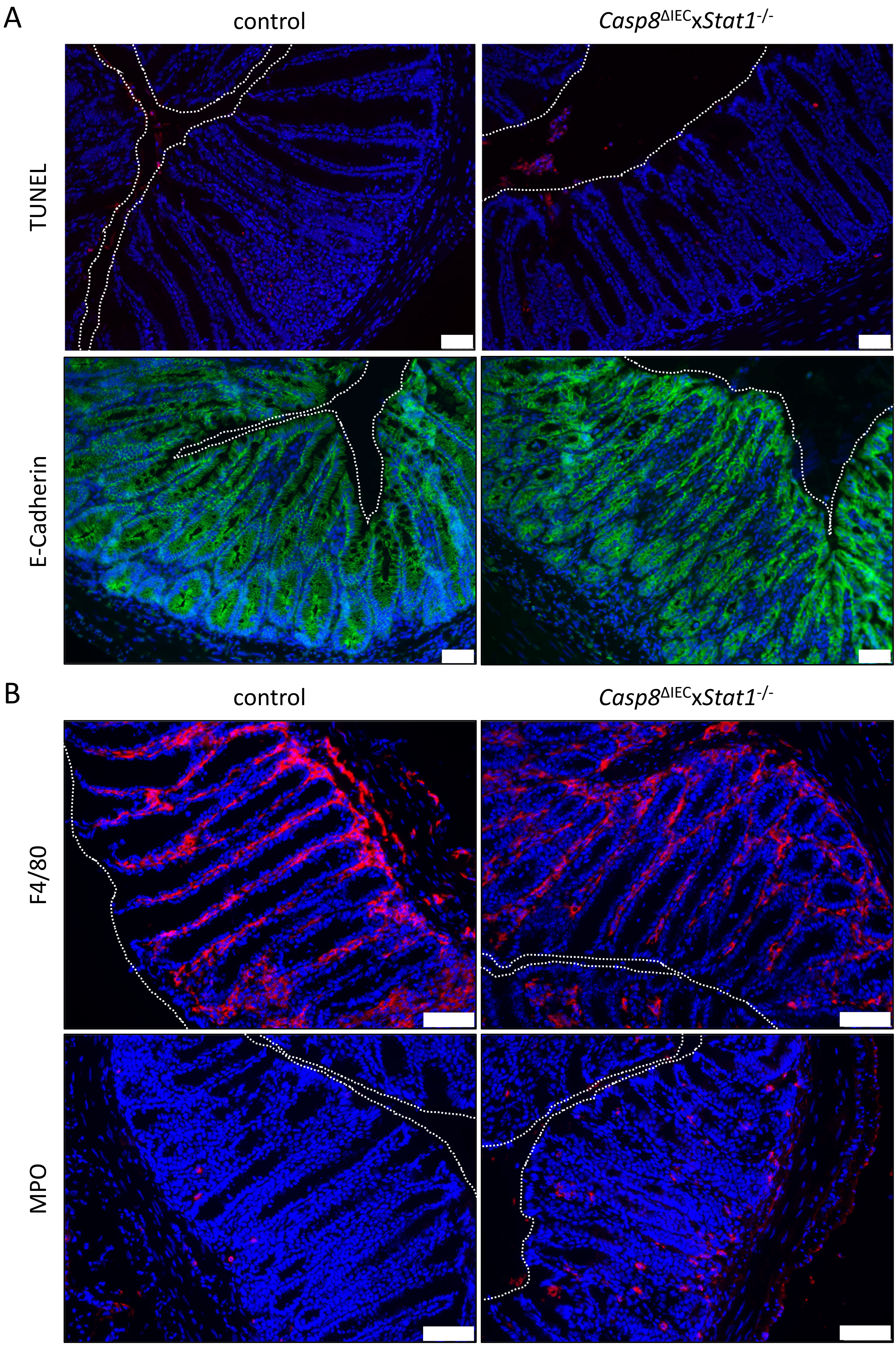
A
control
Casp8ΔIECxStat1-/-
TUNEL
E-Cadherin
B
control
Casp8ΔIECxStat1-/-
F4/80
MPO

Figure S3

Figure S3: *Casp8*$^{\Delta IEC}$x*Stat1*$^{-/-}$ mice display no histological alterations compared to control mice at day 10 post infection

A-B) *Casp8*$^{\Delta IEC}$x*Stat1*$^{-/-}$ mice and control animals were orally infected with *Salmonella* Typhimurium *ΔaroA* and analyzed at day 10 post infection. Pooled data of 3 individual experiments. Representative images of colon cross sections immunohistochemically stained with antibody against E-Cadherin (green, **A**), F4/80 (red, **B**), myeloperoxidase staining (MPO, red, **B**) or stained with TUNEL assay (red, **A**). Nuclei were counterstained with Hoechst 33342 (blue) (scale bar: A 50 µm; B 75 µm).

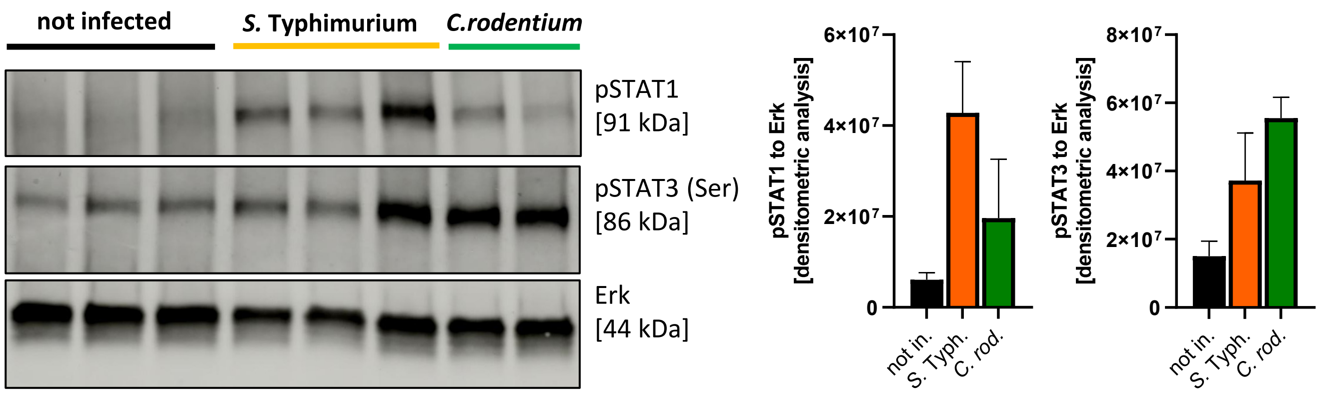

not infected
S. Typhimurium
C.rodentium
pSTAT1
[91 kDa]
pSTAT3 (Ser)
[86 kDa]
Erk
[44 kDa]
pSTAT1 to Erk
[densitometric analysis]
6×10^7
4×10^7
2×10^7
0
not in.
S. Typh.
C. rod.
pSTAT3 to Erk
[densitometric analysis]
8×10^7
6×10^7
4×10^7
2×10^7
0
not in.
S. Typh.
C. rod.

Figure S4

Figure S4: Phosphorylation of STAT1 and STAT3 during *Salmonella* Typhimurium and *Citrobacter rodentium* infection

A-B) Control animals were infected with *S.* Typhimurium *ΔaroA* or *C. rodentium* and sacrificed at day 3 post infection. Western Blot analysis of colonic tissue with antibodies against pSTAT1 (Tyr) and pSTAT3 (Ser). Erk was used as loading control. Densitometry analysis for quantification.

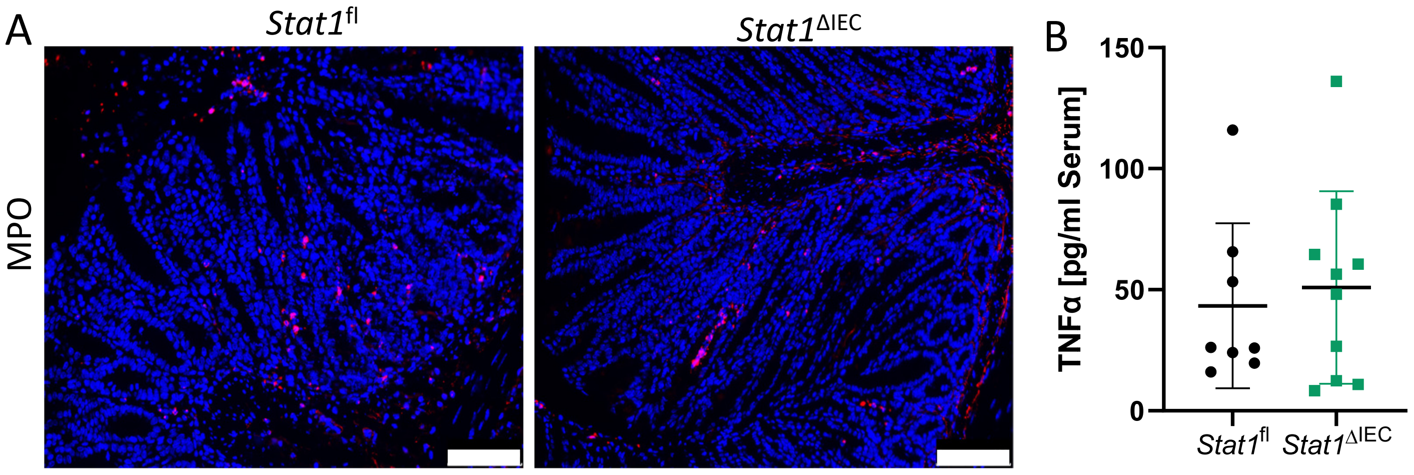
A
*Stat1*fl
*Stat1*ΔIEC
MPO
B
150
100
50
0
TNFα [pg/ml Serum]
*Stat1*fl
*Stat1*ΔIEC

Figure S5

Figure S5: Epithelial STAT1 signaling does not alter immune cell infiltration or TNF level

A-B) *Stat1*$^{\Delta IEC}$ mice and control littermates were orally infected with *Salmonella* Typhimurium (SL1344) and analyzed at day3 post infection. Pooled data of individual experiments (n≥4). A) Representative images of colon cross sections immunohistochemically stained with antibody against myeloperoxidase staining (MPO, red; scale bar: 100 µm). Nuclei were counterstained with Hoechst 33342 (blue). B) TNF serum ELISA. Error bars indicate +/- SD

Supplementary table 1

Antiboy	Cat. No.	Manufacture	Application
FITC Mouse Anti- E-Cadherin	612130	BD	IHC
Anti-Myeloperoxidase antibody	ab9535	abcam	IHC
Phospho-Stat1 (Tyr701) (58D6) Rabbit mAb	9167	Cell Signaling	IHC, WB
Phospho-Stat3 (Tyr705) (D3A7) XP® Rabbit mAb	9145	Cell Signaling	IHC, WB
Anti-beta Actin antibody [AC-15] (HRP)	ab49900	abcam	WB
Anti-rabbit IgG, HRP-linked Antibody	7074	Cell Signaling	WB
Biotin-SP Goat Anti-Rabbit IgG	111-065-144	Jackson ImmunoResearch	IHC
Biotin Goat Anti-Rat Ig	554014	BD Pharmingen	IHC
Erk1/2 (p44/42MAPK)	9102	Cell Signaling	WB
Claudin4	36-4800	Invitrogen	IHC
ZO-1	61-7300	Invitrogen	IHC
Phospho-Stat3 (Ser727)	9134	Cell Signaling	WB
cl. Caspase-8	8592	Cell Signaling	WB
F4/80 Monoclonal Antibody	14-4801-85	eBioscience	IHC

Supplementary table 2

**Gene**	**QuantiTect Primer Assay**	**Cat. No.**
S100a9	Mm_S100a9_1_SG	QT00105252
Reg3g	Mm_Reg3g_1_SG	QT00147455
Villin	Mm_Vil1_1_SG	QT00172935
Mlkl	Mm_Mlkl_1_SG	QT01069285
Tnf	Mm_Tnf_1_SG	QT00104006
Caspase-8	Mm_Casp8_1_SG	QT00171437
Caspase-3	Mm_Casp3_2_SG	QT01164779
Caspase-1	Mm_Casp1_1_SG	QT00199458
Gsdmc4	Mm_Gsdmc4_1_SG	QT00292817
Ripk1	Mm_Ripk1_1_SG	QT00175371
Ripk3	Mm_Ripk3_1_SG	QT01037778
Naip6	Mm_Naip6_1_SG	QT00147721
Nos2	Mm_Nos2_1_SG	QT00100275
Ifnb	Mm_Ifnb1_1_SG	QT00249662
Ifnl2	Mm_Ifnl2_1_SG	QT00319620
Gene	**Sequence**	
Zbp1	TGAGCTATGACGGACAGACG<br>CCGGATTGTGCTGACAAATA	
Nlrp3	AAGTAAGGCCGGAATTCACC<br>AAAATGCCTTGGGAGACTCA	
Caspase-11	TCCTTTAATTTCAGTACATTGCTTTG<br>TGAGGCTTTTTCTCATGGCT	
Gsdmd	CCGGGTTGAGCAGACAATAG<br>ACCACTTTCTCAAAGGCCG	
Gapdh	TCACCACCATGGAGAAGGC<br>GCTAAGCAGTTGGTGGTGCA	
